# Whole Blood Transcriptome Profiling Reveals Positive Effects of Olive Leaves-Supplemented Diet on Cholesterol in Goats

**DOI:** 10.3390/ani11041150

**Published:** 2021-04-17

**Authors:** Andrea Ianni, Francesca Bennato, Camillo Martino, Martina Colapietro, Giuseppe Martino

**Affiliations:** 1 Faculty of Bioscience and Technology for Food, Agriculture and Environment, University of Teramo, 64100 Teramo, Italy; aianni@unite.it (A.I.); fbennato@unite.it (F.B.); mcolapietro@unite.it (M.C.); 2 Istituto Zooprofilattico Sperimentale dell’Abruzzo e del Molise “G. Caporale”, Via Campo Boario, 64100 Teramo, Italy; c.martino@izs.it

**Keywords:** olive leaves, transcriptomics, RNA-seq, cholesterol biosynthesis, dairy goats

## Abstract

**Simple Summary:**

The aim of this study was to analyze the whole blood transcriptome of lactating goats fed a dietary supplementation with 10% olive leaves, one of the main by-products deriving from the olive oil chain supply. This evaluation was effective in identifying the differential regulation of the gene coding for apolipoprotein B mRNA editing enzyme catalytic subunit 2 (APOBEC2), which showed downregulated in goats that received the dietary supplementation. Taking into account the strong association between plasma apoB and low-density lipoprotein, an evaluation was performed of both blood and milk cholesterol. The obtained data demonstrated a significant lower concentration of circulating cholesterol and cholesterol released into the milk through the mammary gland, demonstrating positive effects of olive leaves feeding on animal welfare and potential health benefits for consumers.

**Abstract:**

Agro-industrial by-products represent an important source of compounds credited with high biotechnological potential. In the last decade, considerable interest has developed toward the use of these matrices as dietary supplements in the zootechnical field, paying particular attention to the qualitative aspects associated with animal products. However, less is known about the effect of these matrices on gene expression and thus on animal metabolism. Therefore, the aim of this study was to analyze the whole blood transcriptome of lactating goats fed a dietary supplementation with 10% olive leaves (OL), one of the main by-products deriving from the olive oil chain supply. By applying a false discovery rate (FDR) < 0.05 and a Log2 Fold change (Log2Fc) lower than −0.5 or higher than +0.5, it was possible to identify the differential regulation of gene coding for the apolipoprotein B (apoB) mRNA editing enzyme catalytic subunit 2 (APOBEC2), which showed downregulation in goats that received the dietary supplementation. An evaluation of both blood and milk cholesterol was performed, taking into account the strong association between plasma apoB and low-density lipoprotein (LDL). Results showed significantly lower concentrations of circulating cholesterol and cholesterol released into the milk through the mammary gland, demonstrating positive effects of OL feeding on animal welfare and potential health benefits for consumers.

## 1. Introduction

The olive oil chain supply is responsible for the production of waste residues whose disposal represents a pivotal issue both from an environmental and economic point of view. This problem is obviously strongly felt in Mediterranean countries, where we observe a marked production of olive oil and non-negligible amounts of related by-products are therefore accumulated, specifically represented by olive pomace and wastewaters, but also leaves and other plant pruning residues [1,2]. As for other agro-industrial sectors, the strategy of valorizing these by-products is now well established, trying to identify alternative uses justified by the high richness of these plant matrices in bioactive compounds [3,4]. From this point of view, much has been done in the zootechnical field, through the development of feeding strategies based on the use of these by-products, both for ruminants and monogastrics [5,6,7]. Specifically for olive oil by-products, over time, considerable information has been collected on the effects induced on both the qualitative and quantitative aspects of animal products. Most studies in both meat and dairy products have specifically shown an increase in concentration of polyunsaturated fatty acids and greater oxidative stability, with significant implications in the improvement of the health functionality and products’ shelf-life [8,9,10].

With specific regard to olive leaves (OL), reference is made to a matrix well characterized for the high content of phenolic compounds, mainly oleuropeosides, flavonoids, and phenolic acids, which confer a high anti-inflammatory and antioxidant potential [11]. For that reason, in the last decade, OL have found widespread application in animal nutrition, especially for dairy ruminants. In the review presented by Molina-Alcaide and Yáñez-Ruiz [12], this by-product was presented as fibrous with a low digestibility, especially of crude protein, as well as capable of negatively influencing microbial protein synthesis and fermentation in rumen. A significant improvement in the milk fatty acid profile was however evidenced in lactating animals compared with animals fed conventional diets. More recently, the efficacy of an OL-supplemented diet in inducing an increase in concentration of linolenic acid in goat milk was confirmed; furthermore, an improvement in the oxidative stability of derived dairy products was observed, probably as a consequence of the reduction of lipolytic events during storage and ripening [13,14,15].

All these findings represent a stimulus to the use of OL as an ingredient in the diet of lactating ruminants in order to obtain milk and cheese improvements from a nutritional point of view. However, little or nothing is known about the effect of this feeding strategy on the animal transcriptome. The use of experimental diets based on the integration of plant matrices rich in bioactive compounds, as in the case of OL, should positively contribute to the regulation of gene expression in dairy ruminants. This approach has allowed other studies to highlight the effect of diet on the molecular mechanisms responsible for variations observed not only on productive traits, but also in aspects related to animal health [16,17,18]. Therefore, the specific objective of this study was to analyze the whole blood transcriptome of goats by using an RNA-sequencing approach, considering the hypothesis that a dietary supplementation with OL could be effective in modifying the host’s metabolic pathways. It should not be underestimated that this can also represent a useful point of view for optimizing nutrition protocols for farm animals.

## 2. Materials and Methods

The study was conducted in a commercial company that, during the spring months, habitually integrates the diet of dairy goats with OL and other residues coming from pruning. For this reason, no breeding practices other than those normally adopted have been introduced. Additionally, the trial has been planned and executed by taking into account the Directive 2010/63/EU of the European Parliament (European Union, 2010) and Directive 86/609/EEC (European Economic Community, 1986), which deals with the protection of animals used for scientific purposes [19,20]. Blood sampling was performed by authorized veterinarians concurrently with planned blood withdrawal for the brucellosis prophylaxis; therefore, no ethical declaration is necessary.

### 2.1. Experimental Plan, Animals and Diets

This study is part of a wider experimentation concerning the effect of an OL-supplemented diet on lactating goats, with the purpose of focusing the attention on both the qualitative characteristics of milk and cheese and the biochemical and molecular aspects influenced by the diet. For this reason, the experimental design has already been described [15]. Briefly, the trial lasted 30 days and involved 30 Saanen goats homogeneous for age (46 ± 2 months), weight (52.7 ± 4.3 kg), lactation days (86 ± 7 days in milk), milk yield (2296 ± 281 g/day), and a body condition score (BCS) equal to 2.78 ± 0.18.

At the beginning of the experimentation, the animals were evenly separated into two groups that were indoor housed in two adjacent but separate areas characterized by a space shared by goats belonging to the same group, a drinking trough, and provisional single bunks on straw, useful for the individual administration of the diets. Each goat received polyphite daily hay ad libitum, while twice daily (in the morning and in the evening) was administered a custom-formulated concentrate (a total of 1 kg/head) whose ingredients and chemical composition have been previously reported [15]. The control group (CTR) was fed a standard diet that was formulated by taking into account the nutritional needs of lactating goats, while the experimental group (OL+) received the same basic diet but supplemented with 10% OL on a dry matter (DM) basis.

### 2.2. Milk and Blood Samples Collection

At the end of the experimental period, individual milk samples were collected from all the animals involved. Milk samples were then immediately aliquoted and stored at −20 °C until the time of analysis.

Additionally, in the case of whole blood (WB), sampling was performed at the end of the 30 days of dietary supplementation on a total of 20 animals randomly selected, 10 from the CTR and 10 from OL+. In order to evaluate the blood biochemical parameters, WB was collected in Venoject glass tubes containing ethylenediaminetetraacetic acid (EDTA) or sodium heparin (Terumo Italia, Rome, Italy). Samples were immediately cooled, and plasma separation took place within 30 min of collection. Following a centrifugation step at 500 RCF for 15 min, the obtained samples were stored at −20 °C until analysis.

Although a larger number of animals was used in the study, for the RNA-Seq analysis, sampling was done by drawing 2.5 mL of blood from the jugular vein on a total of 10 goats randomly selected among those already subjected to WB sampling for biochemical evaluations (5 from the CTR and 5 from OL+). Duplicate WB samples from each of the selected animals were collected in PAXgene™ tubes (Qiagen SpA, Milan, Italy), stored overnight at room temperature and then at −20 °C until RNA extraction, as prescribed by the manufacturer.

### 2.3. Blood Analysis

Blood samples obtained from CTR (*n* = 10) and OL+ (*n* = 10) animals were analyzed for blood cell count with leukocyte formula (total white blood cells, lymphocyte, monocyte, neutrophils, eosinophils, and basophils). Analysis was performed by using a laser-based hematology analyzer with software applications for animal species (ADVIA 120 hematology system, Siemens, Munich, Germany). Serum samples were instead evaluated by using an automatic biochemistry analyzer (ILAB 650, Instrumentation Laboratory-Werfen, Milan, Italy), in order to quantify different target compounds (triglycerides, glucose, cholesterol, calcium, urea, aspartate aminotransferase (ASAT), alanine aminotransaminase (ALAT)).

### 2.4. Cholesterol Evaluation in Milk

Cholesterol quantification was performed in raw milk samples obtained from the same animals involved in blood sampling (*n* = 10 from CTR and *n* = 10 from OL+). The analysis was carried out by applying the method previously described by Oh et al. [21] with slight modifications. In total, 1 mL of milk was transferred into a 15 mL tube and subjected to saponification by adding 1 mL of 10% KOH in ethanol (w/v) for 30 min at 70 °C. The addition of 5 mL of diethyl ether and 2 mL of H_2_O therefore allowed us to proceed with the extraction of the unsaponifiable fraction. The extraction was repeated 3 times, and the diethyl ether extract was transferred into a 50 mL round-bottomed flask and dried with a Strike-Rotating Evaporator (Steroglass s.r.l., Perugia, Italy) with the bath temperature set at 50 °C. The sample was then recovered with 1 mL of methanol, and a 20 µL aliquot was directly injected into the HPLC. Cholesterol identification was achieved through a HPLC chromatographic system (Varian, Harbor City, CA, USA) equipped with a Supelcosil LC-18 HPLC column (25 cm × 4.6 mm, 5 μm; Sigma-Aldrich, Milan, Italy). Isocratic conditions with a mobile phase containing 75% acetonitrile and 25% methanol were used. The flow rate of the mobile phase was 1.5 mL/min, and the column temperature was set at 38 ± 0.1 °C. The cholesterol peak was detected at 205 nm. Cholesterol (Sigma-Aldrich, Milan, Italy) was used as standard in order to obtain a calibration curve that was linear in the range of concentration from 0.01 to 0.50 mg/mL (R^2^ = 0.983). The obtained regression lines were exploited to calculate the amount of cholesterol that was expressed in mg/mL.

### 2.5. Library Preparation and RNA-Seq Analysis

The RNA-Seq experiments, inclusive of the RNA extraction and bioinformatics evaluations, were performed by an external company (Genomix4life SRL, Baronissi, Salerno, Italy). Total RNA extraction, library preparation, and sequencing were performed by following the procedure already described by Iannaccone et al. [18].

The quality control of the obtained raw sequences (fastq files) was performed by exploiting the FastQC tool (Version 0.11.8) for high throughput sequence data, available on http://www.bioinformatics.babraham.ac.uk/projects/fastqc (accessed on 12 October 2020) [22]. The bioinformatic tool cutadapt (version 2.5) [23] was then used in order to remove the adapter sequences and the very short reads (reads length < 20). The sample was mapped on reference *Capra hircus* genome (ARS1) using the bioinformatics tool STAR (version 2.7.5c) [24], by referring to the standard parameters for paired reads. The reference track was the assembly *Capra hircus* (ARS1, INSDC Assembly GCA_001704415.1) obtained from Ensembl (release 101—August 2020—EMBL-EBI) [25].

The featureCount algorithm (version 2.0) [24] was then used to quantify the expressed transcripts for each replicate of sequenced samples. A false discovery rate (FDR) less or equal to 0.05 (FDR ≤ 0.05) was considered in order to identify differentially expressed genes (DEGs). The Bioconductor package DESeq2 [26] was used to normalize the data, using the median of ratio, to perform the differential expression analysis.

### 2.6. Statistical Analysis

Statistical analysis of the obtained data was performed by using the software SigmaPlot 12.0 (Systat software Inc., San Jose, CA, USA). The ANOVA model was used, and the Student’s *t*-test was applied for means comparison. *p*-values lower than 0.05 have been associated with the presence of significant differences.

## 3. Results

### 3.1. Hematochemical Characterization

**Figure 1:**
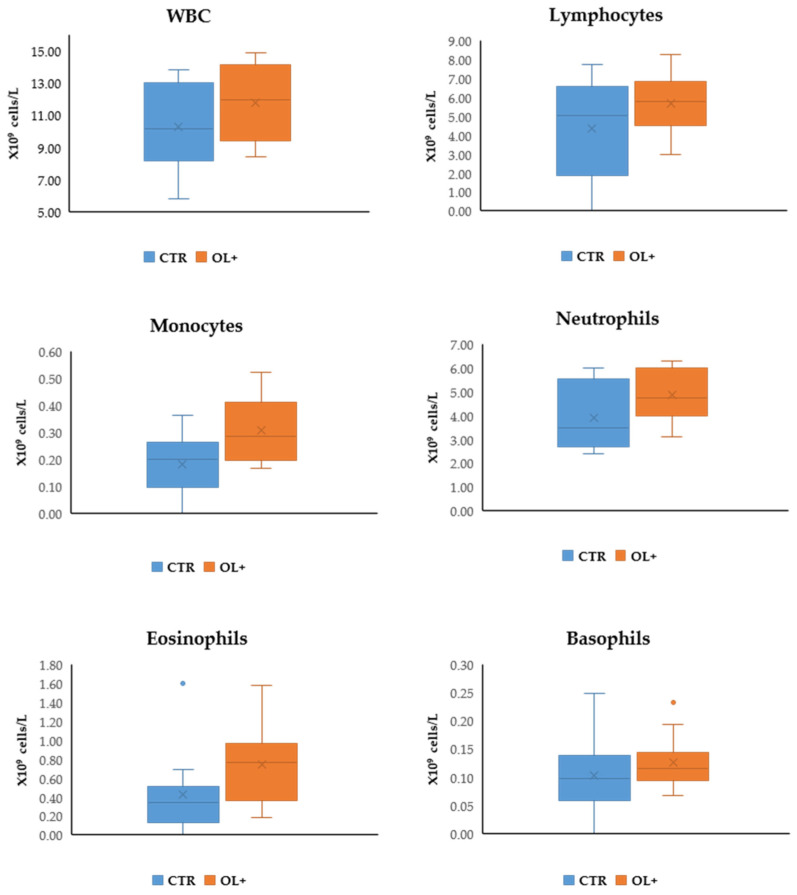
Effect of the dietary olive leaves (OL) supplementation on blood cells count. Differences were analyzed through the Student’s *t*-test. Data are reported as mean values ± standard deviation. (WBC: white blood cell; CTR: control group, *n* = 10; OL+: experimental group, *n* = 10).

The complete blood cell count and main biochemical parameters in serum samples were evaluated in order to characterize the general health status of animals fed the dietary OL supplementation. No significant variations (*p* > 0.05) were evidenced in the cell count of the different considered families (Figure 1).

**Figure 2:**
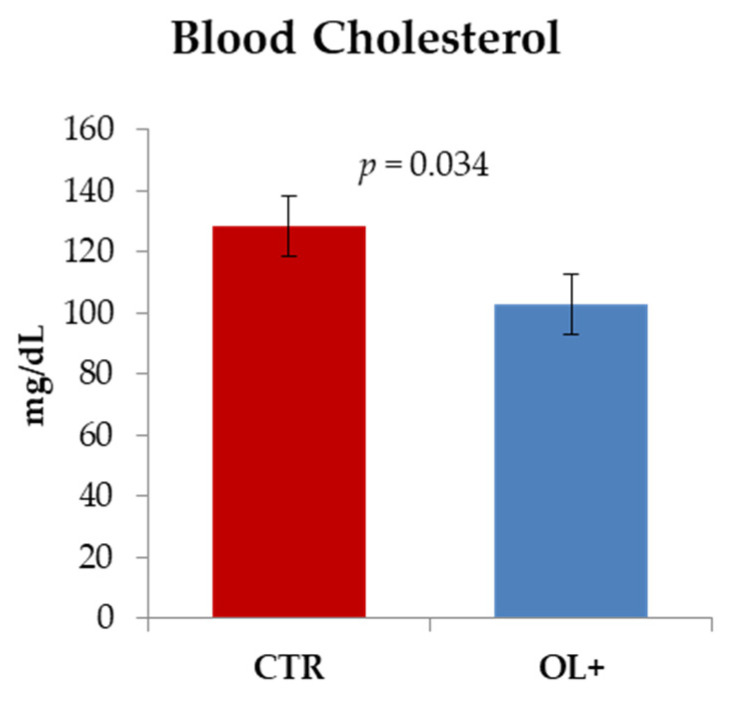
Effect of the dietary olive leaves (OL) supplementation on cholesterol content in blood samples. Data are reported as mean values (mg/dL) ± standard deviation. Differences were analyzed through the Student’s *t*-test (CTR: control group, *n* = 10; OL+: experimental group, *n* = 10).

The only significant difference was instead observed in serum (Figure 2), in which a lower concentration of cholesterol was recorded in the OL+ samples compared to those obtained from animals fed the standard diet (128.5 ± 25.7 mg/dL vs. 102.9 ± 17.2 mg/dL in CTR and OL+, respectively; *p* < 0.05). No differences were highlighted for triglycerides, glucose, calcium, urea, ASAT, and ALAT (data not shown).

### 3.2. Cholesterol Amount in Goat Milk

**Figure 3:**
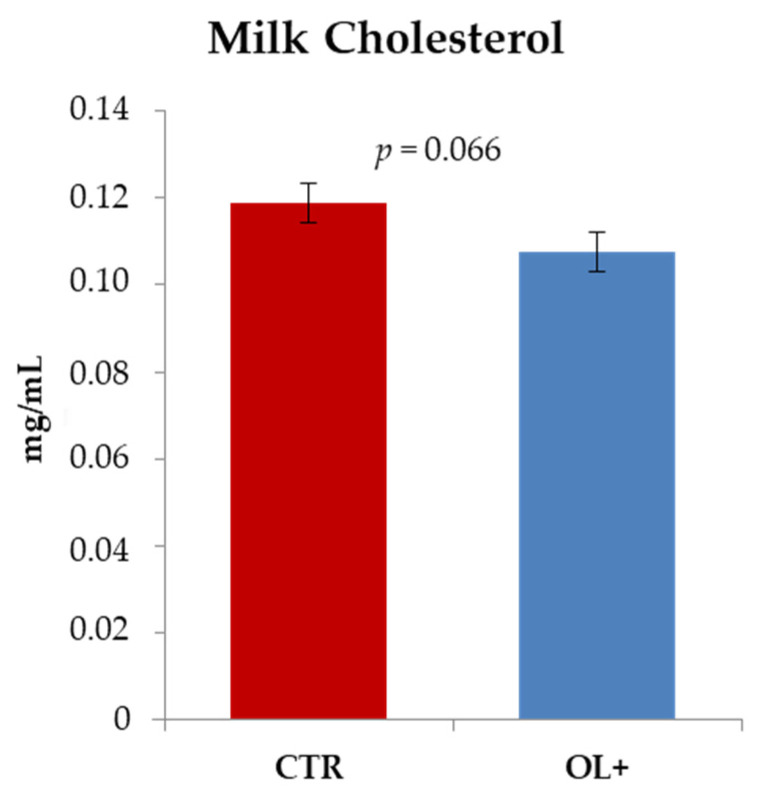
Effect of the dietary olive leaves (OL) supplementation on cholesterol content in milk samples. Data are reported as mean values (mg/mL) ± standard deviation. Differences were analyzed through the Student’s *t*-test (CTR: control group, *n* = 10; OL+: experimental group, *n* = 10).

The administration to goats of a diet supplemented with 10% OL (on a DM basis) did not induce changes in the cholesterol content of milk (Figure 3), although it should be reported that the *p*-value associated with this variation showed a trend toward significance as it was lower than 0.1 (0.119 ± 0.009 mg/mL vs. 0.108 ± 0.008 mg/mL in CTR and OL+ samples, respectively; *p* = 0.066).

### 3.3. Influence of Dietary OL Supplementation on Whole Blood Transcriptome

The sequencing of RNA extracted from whole blood samples (CTR, *n* = 5; OL+, *n* = 5), allowed us to analyze the transcriptomic signature in dairy goats that received the OL dietary supplementation. By applying a false discovery rate (FDR) < 0.05 combined with a log2 fold change (log2FC) higher than 0.5 or lower than −0.5, only a differentially expressed gene (DEG) was identified. Specifically, in OL+ animals, there was evidence of a downregulation in the expression of Apolipoprotein B mRNA editing enzyme catalytic subunit 2 (APOBEC2; NCBI Gene ID: 102186351; FDR = 0.008; Fold Change = −47.636906).

A matrix of all genes expressed in all samples with the corresponding read-counts was created, and data were then normalized by using the median of ratio, to perform the differential expression analysis. In particular, the counts were divided by sample-specific size factors determined by median ratio of gene counts relative to geometric mean per gene.

## 4. Discussion

In the last decade, experimentations concerning the use of agri-food by-products as dietary ingredients for farm animals had a significant boost mainly due to the need to valorize these plant matrices, the disposal of which represents both an environmental and economic issue. In the specific case of OL as a feeding ingredient for dairy ruminants, several studies have been conducted, which, even recently, have characterized the aspect concerning the potential effects on the quality of milk but also fresh and ripened cheeses [13,14,15]. To our knowledge, there is, however, a lack of information regarding the effect of this feeding strategy on animal metabolism, an aspect that can contribute to obtain clarifications about variations observed in the nutritional qualities of obtained dairy products, as well as on the animals’ health.

In this study, we decided to investigate the peripheral blood of goats fed a dietary OL supplementation, since in previous nutrigenomic studies on ruminants, it was possible to identify differentially expressed genes with extreme sensitivity and accuracy [16,18,27]. In addition to this, the characterization of gene expression carried out on blood tissue allows us to collect information capable of reflecting the molecular and biochemical mechanisms occurring in other tissues or organs [28]. Specifically, the main finding of this study concerns the fact that dietary OL supplementation was shown to be effective in modifying the gene expression of lactating Saanen goats. This datum is therefore in a condition of affinity with what has already been shown in recent studies in which the enrichment of ruminants’ diet with plant matrices led to changes in the transcription process of specific genes [16,18,27]. The aspect that in any case must be immediately highlighted concerns the fact that generally, the RNA-seq approach on whole blood is capable of highlighting variations of expression that involve entire gene clusters and not only a few transcripts or even one, just like in our case. This aspect therefore deserves to be further investigated to verify whether this finding can be confirmed or enriched by identifying other molecular targets influenceable by the administered feeding strategy. In any case, to our knowledge, this study represents the first whole blood transcriptome profiling of goats fed a dietary OL supplementation.

Analyses concerning blood cell count and the evaluation of specific parameters in serum samples were performed in order to obtain a preliminary assessment of the health status of the animals involved in the trial, and whether the diet had somehow induced significant effects from this point of view. In the counts of the various cell populations, no significant differences were found, while OL administration seems to have been effective in inducing a reduction in blood cholesterol. Although in this case, it is not possible to perform a comparison with similar trials, it may however be useful to refer to the study conducted by Olmez et al. [29], who evaluated the effect of an olive leaf extract in rats fed a cholesterol-enriched diet. Administration of the extract was shown to be effective in reducing cholesterol and LDL-cholesterol levels in blood, a datum therefore associated with a lower predisposition to the onset and progression of atherosclerosis. Similarly, a phenolic-rich extract obtained from olive mill wastewaters induced hypocholesterolemic effects in rats fed a cholesterol-rich diet. In this case, the finding was also correlated with broader spectrum assessments aimed at determining lipid peroxidation and antioxidant enzymatic activities in heart, liver, kidney, and aorta. Specifically, an improved lipid oxidative stability was found in the analyzed tissues, together with an increase in the catalytic activities mediated by catalase and superoxide dismutase. The study performed by Iannaccone et al. [30], in which laying hens received a dietary supplementation with olive pomace with consequent modulation of biochemical pathways related to cholesterol biosynthesis (a finding that was consistent with the reduction in egg yolk cholesterol), also fits into this area. As regards ruminants, there is a lack of specific references to the effect of the dietary administration of olive by-products in the modulation of blood parameters, and in cases where this was done, no consideration was given to cholesterol. For instance, in the study presented by Obeidat [31], the effects induced on Awassi lambs as a consequence of a feeding strategy based on the use of an olive cake supplementation were characterized. In that case, only an increase in the amount of serum glucose was highlighted, while no variations were evidenced for urea, aspartate aminotransferase, alanine aminotransferase, and alkaline phosphatase.

The results obtained from the blood analysis led to also considering the data concerning the cholesterol content in milk. Very interesting is the fact that such a parameter was found to be lower in OL+ milk samples, with a trend toward statistical significance (*p* = 0.066). The presence of limited concentrations of cholesterol in foods is of great interest from a nutritional point of view because high levels of this compound in human plasma correlate with an increasing risk of cardiovascular disease. Moreover, in the presence of specific conditions, especially following heat treatments [32], cholesterol in food can undergo oxidation generating products, namely oxysterols, which are in turn very harmful to human health [33]. In this regard, a comparison is useful with what has been previously reported by Gómez-Cortés et al. [34], who explored alternative strategies to decrease cholesterol content in sheep milk cheeses. Specifically, authors highlighted that feeding animals a diet rich in specific polyunsaturated fatty acids was effective in reducing the cholesterol content in milk. In particular, dietary supplementation was performed by including extruded linseed in the ration. As is known, linseed is rich in polyunsaturated fatty acids, with relevant concentrations of linolenic acid (C18:3 cis-9, cis-12, cis-15), which also represent the major fatty acid detectable in the OL used in this trial [15]. Contrary to what has just been reported, no changes in milk cholesterol were observed in the study conducted by Symeou et al. [35], in which lactating Chios ewes were fed diets supplemented with ensiled olive cake. In this case, the only noteworthy changes concerned the reduction of saturated fatty acids and an increase in concentration of fatty acids of interest for consumers’ health, such as conjugated linoleic acids and linoleic acid.

The sequencing of the transcripts isolated in whole blood revealed a downregulation in the expression of APOBEC2 in goats fed the dietary OL supplementation. This protein belongs to the wide family of apolipoprotein B messenger RNA-editing enzyme catalytic polypeptides, which are able to deaminate mRNA and, in specific conditions, single-stranded DNA [36]. Specifically, this protein family is easily identifiable through the amino acid similarity found at the level of the catalytic zinc-dependent domain, responsible for cytidine or deoxycytidine deamination [37].

The best known and characterized member of this group of enzymes is certainly APOBEC-1, which is involved in apolipoprotein B (apoB) RNA editing. Specifically, apoB is reported to be present in mammals in two distinct forms, apoB100 and apoB48; apoB100 is secreted in the liver, while apoB48 is obtained in the small intestine following the deamination of a cytidine base in the nuclear apoB transcript, with the consequent introduction of a translational stop codon. Overall, apoB represents a fundamental element in the assembly and secretion process of lipid structures, including triglycerides and cholesterol of both dietary and endogenous origin. Furthermore, apoB glycoproteins also mediate the intravascular transport and cellular uptake of different lipoproteins; therefore, the importance of apoB is simultaneously associated with processing, absorption, and regulation of circulating lipoproteins [38]. With specific regard to this last function, recent findings allowed us to hypothesize that apoB concentration could be useful in providing a direct measure of the number of circulating atherogenic lipoproteins. This position was advanced following the analysis of epidemiological data which attributed a greater importance to apoB than cholesterol alone as a risk index for the onset of vascular diseases [39].

The specific function of APOBEC2 in mammals, and especially in ruminants, has not been fully characterized; however, a discrete sequence homology with APOBEC1 has been highlighted [40], and therefore, it is not excluded that this enzyme may contribute to the apoB secretion. A significant reduction in the expression of this enzyme could therefore have positive effects on lipid metabolism, at least partially justifying the encouraging results obtained in this study in the assay of blood and goat milk cholesterol.

## 5. Conclusions

This is the first study to report whole-transcriptome profiling of lactating Saanen goats fed a dietary OL supplementation. The obtained findings suggest the downregulation of a gene encoding for a member of the APOBEC family. This condition seems to correlate with a reduction in blood cholesterol and a tendency for the compound to accumulate in lower concentrations in the produced milk, thus generating a condition consistent with an improvement of both animal welfare and the potential health benefits for consumers.

## Data Availability

The data reported can be made available by the corresponding author following reasonable request.
